# Does dog-ownership influence seasonal patterns of neighbourhood-based walking among adults? A longitudinal study

**DOI:** 10.1186/1471-2458-11-148

**Published:** 2011-03-04

**Authors:** Parabhdeep Lail, Gavin R McCormack, Melanie Rock

**Affiliations:** 1 Population Health Intervention Research Centre, Calgary Institute of Population and Public Health, University of Calgary, Alberta, Canada

## Abstract

**Background:**

In general dog-owners are more physically active than non-owners, however; it is not known whether dog-ownership can influence seasonal fluctuations in physical activity. This study examines whether dog-ownership influences summer and winter patterns of neighbourhood-based walking among adults living in Calgary, Canada.

**Methods:**

A cohort of adults, randomly sampled from the Calgary metropolitan area, completed postal surveys in winter and summer 2008. Both winter and summer versions of the survey included questions on dog-ownership, walking for recreation, and walking for transportation in residential neighbourhoods. **Participation **in neighbourhood-based walking was compared, among dog-owners and non-owners, and in summer and winter, using general linear modeling. **Stability **of participation in neighbourhood-based walking across summer and winter among dog-owners and non-owners was also assessed, using logistic regression.

**Results:**

A total of 428 participants participated in the study, of whom 115 indicated owning dogs at the time of both surveys. Dog-owners reported more walking for recreation in their neighbourhoods than did non-owners, both in summer and in winter. Dog-owners were also more likely than non-owners to report participation in walking for recreation in their neighbourhoods, in summer as well as in winter. Dog-owners and non-owners did not differ in the amount of walking that they reported for transportation, either in summer or in winter.

**Conclusions:**

By acting as cues for physical activity, dogs may help their owners remain active across seasons. Policies and programs related to dog-ownership and dog-walking, such as dog-supportive housing and dog-supportive parks, may assist in enhancing population health by promoting physical activity.

## Background

Participation in regular physical activity is important for the prevention of many chronic diseases, including type 2 diabetes, hypertension, cardiovascular disease, obesity, osteoporosis, some cancers, irritable bowel syndrome, dementia and depression [1-5]. Despite these health benefits, at least 60% of the global population does not achieve the minimum recommendation of 30 minutes of moderate-intensity physical activity daily [6]. In Canada, only 31% of adults walk more than one hour per week [7], and in the Province of Alberta, 25% of adults report not walking for leisure at least once in the previous three months [8]. The correlates of physical activity include demographic, biological, psychological, social, and physical environmental factors [9-12]. Moreover, physical activity behaviour tends to fluctuate across seasons, especially in countries where extreme differences in seasonal weather patterns are experienced [13-15]. For example, levels of physical activity are significantly lower during colder months particularly in countries that experience extreme weather such as the United States, Canada, Scotland, The Netherlands, and France [15]. During winter, 64% of Canadians are inactive as compared with 49% in the summer [13]. Extended periods of physical inactivity results in physiological deconditioning and, when accumulated over time, could increase the risk of adverse health outcomes [16-18].

Several quantitative studies have found that dog-owners tend to be more physically active than non-owners [11,19]; all of these cross-sectional studies, however, were conducted in geographical areas with mild or moderate climates, including one Canadian study [19] undertaken in a coastal city with a temperate climate. Focus group research, meanwhile, suggests that dog-owners view their dogs as a source of companionship and motivation for getting outdoors and walking [20,21] - regardless of weather conditions [21]. As one participant put it, "But if you've got a dog, you've got to come out in all weathers" [21], p.443], while another said, "If it's absolutely foul weather sometimes you think, oh dear, I've got to go out, but once you're out there, providing you're dressed appropriately, it's just fabulous, so yes, so I walk every day" [21], p.444]. The available evidence thus suggests a potential role for dog-ownership in overcoming or mitigating the reduction in physical activity often observed during winter months, but this possibility has yet to be examined quantitatively. We investigated the extent to which dog-ownership influences seasonal patterns in neighbourhood-based walking among adults living in highly-variable climate.

## Method

### Sample, Setting and Data Collection

This paper presents longitudinal findings among respondents nested within a larger observational study. A random cross-section of adults (n = 2223) residing in Calgary, Canada participated in telephone-interviews in winter 2007/08 (January to April) as part of the EcoEUFORIA (Economic Evaluation of Using Urban to Increase Activity) project. One adult per household was invited to participate in the telephone-interview. To be eligible to participate, respondents had to reside in the Calgary metropolitan area, be 18 years or older, and be proficient in English. Details regarding the telephone survey are fully described elsewhere [7]. The Conjoint Health Research Ethics Board at the University of Calgary approved this study.

The Calgary metropolitan area is 745 km^2^, includes 1071515 residents and 122325 dog-owning households [22] and offers a relatively high standard of living [23]. Located east of the Rocky Mountains, Calgary is elevated over one-kilometer above sea level, and has a continental climate. Calgary winters are characterized by subzero temperatures, snow fall, and periodic daytime thaws followed by overnight freezing, resulting in icy conditions. Summers in Calgary, meanwhile, are characterized by warm temperatures with some precipitation and occasional heat waves. The amount of daylight also varies considerably from summer to winter. At winter solstice (21 December), sunset occurs 7.9 hours after sunrise, whereas at summer solstice (21 June), sunset occurs 16.56 hours after sunrise [24]. During the winter data collection (January to April 2008), the mean temperature ranged from -8.5 to 1.5 degrees Celsius, with total snowfall ranging from 16.8 to 65.8 cm per month, respectively [25]. During the summer data collection (August, 2009), the mean temperature was 15.9 degrees Celsius, with a total rainfall of 62.2 mm [25].

**Figure 1:**
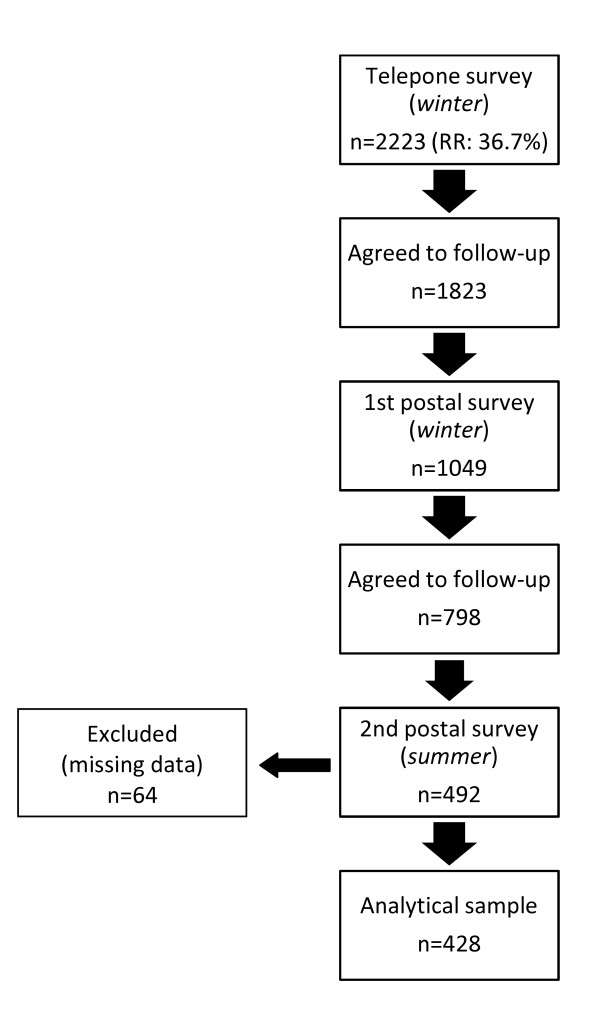
**Summary of the sampling method and respondent participation**.

Of those who completed the telephone-interview, 1823 respondents agreed to complete a postal questionnaire, which was sent out within one week of completing the telephone interview. Postal questionnaires measured, among other variables, demographic characteristics (income, education, age, sex, housing tenure and housing type), health behaviours, and dog-ownership [7]. Overall 1049 respondents completed and returned the postal questionnaire (by April 2009). Of those, 798 agreed to participate in a second postal questionnaire sent in summer (August, 2009). The second postal questionnaire included an expanded set of dog-ownership questions, as well as repeating the physical activity items from the telephone survey. A total of 428 respondents completed both the winter and summer questionnaires (Figure 1).

### Measures

#### Demographic characteristics

The winter telephone-interview and questionnaire captured baseline demographic characteristics including age, sex, annual household income (<$60000, 60000-119999, or ≥120000), highest education achieved (≤high school, technical college, undergraduate university, or postgraduate university), marital status (single or married), ethnicity (white or non-white) and housing type (detached/semi-detached or other housing).

#### Neighbourhood-specific walking

The winter telephone-interview and the summer questionnaire included items from the Neighbourhood Physical Activity Questionnaire [26] capturing transportation and recreational walking undertaken inside the neighbourhood during a usual week (everywhere within a 15-minute walk from home). Specifically, respondents were asked: "In a usual week how many times do you walk as a means of transportation such as going to and from work, walking to the store or walking to the bus stop or LRT in your neighbourhood or local area?" Respondents were also asked: "In a usual week how many times do you walk for recreation, health or fitness (including walking your dog) in or around your neighbourhood or local area?" Respondents reporting participation also reported the total time spent walking for transportation or recreation in their neighbourhood. Neighbourhood-specific walking items capturing frequency and duration have been shown to have acceptable reliability [26,27].

#### Dog-ownership

In the winter and summer questionnaires respondents reported the number of dogs their household owned. Respondents living in a household with dogs were coded as dog-owners, as opposed to non-owners. Those that changed their dog-ownership status between the two surveys were not included in the analysis (n = 10).

### Statistical Analysis

All baseline demographic characteristics were compared for dog-owners and non-owners using Pearson's chi-square for categorical variables and independent t-tests for continuous variables. The absolute difference in minutes of NTW and NRW was also calculated (i.e., winter minus summer minutes) with positive values representing more weekly walking in winter compared with summer, and negative values representing less weekly walking in winter compared with summer. A general linear model was used to regress the difference in minutes of NTW and NRW between winter and summer onto dog-ownership status while adjusting for demographic characteristics. To aid with interpretation, estimates associated with dog-ownership status are presented as marginal (i.e., adjusted) means.

To examine whether or not dog-owners and non-owners participation in neighbourhood walking remained stable from winter to summer, respondents were coded into four categories: 1) *consistent participators*: any walking in both winter and summer; 2) *consistent non-participators*: no walking in either winter or summer; 3) *summer-only participators: *some walking in summer, but not in winter; and 4) *winter-only participators*: some walking in winter, but not in summer. Logistic regression odds ratios (OR) and 95% confidence intervals (95%CI) were used to estimate the association between dog-owner status and stability of participation (or non-participation) in neighbourhood-based walking for transportation (NTW) and for recreational (NRW) from summer to winter, adjusting for demographic characteristics. Stata 10.1 statistical software was used for all analyses.

## Results

The average age of the sample (n = 428) was 53 ± 15 years. The sample included 115 dog-owners (26.9%) and 313 non-owners (73.1%). While dog-owners were disproportionately white, higher-income, residing in a detached or semi-detached house, there was no difference between dog-owners and non-owners in education, marital status or sex (Table 1).

**Table 1:** Demographic profile and summer and winter neighbourhood walking behaviour among all respondents (n = 428), owners (n = 151) and non-owners (n = 313)

	All respondents	Non-Owners	Dog-Owners	p-value
	% (n)	% (n)	% (n)	
**Sex**				
Male	35.1 (150)	37.1 (116)	29.6 (34)	.150
Female	64.9 (278)	62.9 (197)	70.4 (81)	
				
**Marital status**				
Single	33.4 (143)	76.2 (109)	23.8 (34)	.307
Married	66.6 (285)	71.6 (204)	28.4 (81)	
				
**Income**				
<$60000	34.3 (147)	38.3 (120)	23.3 (27)	.004*
$60000 -119999	33.9 (145)	33.9 (106)	33.9 (39)	
≥$120000	31.8 (136)	27.8 (87)	42.6 (49)	
				
**Education**				
High school or less	25.6 (110)	26.5 (83)	23.5 (27)	.520
Technical college/school	25.5 (109)	25.2 (79)	26.1 (30)	
University (undergraduate)	33.6 (144)	31.9 (100)	38.3 (44)	
University (graduate)	15.2 (65)	16.3 (51)	12.2 (14)	
				
**Type of residence**				
Detached/semi-detached	74.5 (319)	68.4 (214)	91.3 (105)	.001*
Other	25.5 (109)	31.6 (99)	8.7 (10)	
				
**Ethnicity**				
White	89.9 (385)	87.9 (275)	95.7 (110)	.017*
Non-white	10.1 (43)	12.1 (38)	4.3 (5)	
				
**Neighbourhood walking for recreation**				
Consistent participator (NRW in winter and summer)	65.1 (279)	58.8 (184)	82.6 (95)	.001*
Summer participator (NRW in the summer only)	8.9 (38)	12.1 (38)	0 (0)	.001*
Winter participator (NRW in the winter only)	10.5 (45)	10.5 (33)	10.4 (12)	.974
Consistent non-participator (no NRW in winter and summer)	15.4 (66)	18.5 (58)	7.0 (8)	.003*
				
				
**Neighbourhood walking for transportation**				
Consistent participator (NTW in winter and summer)	43.7(187)	47.0 (147)	34.8 (40)	.024*
Summer participator (NTW in the summer only)	11.9 (51)	11.8 (37)	12.2 (14)	.920
Winter participator (NTW in the winter only)	12.8 (55)	11.8 (37)	15.6 (18)	.294
Consistent non-participator (no NTW in winter and summer)	24.8 (135)	29.4 (92)	37.4 (43)	.114

*Significant difference between dog owners and non-owners (p < .05).

In the summer, dog-owners on average spent 213.6 min/week in NRW, compared with non-owners who spent on average 123.3 min/week (Table 2). Meanwhile, in the winter, dog-owners on average spent 253.2 min/week in NRW compared with non-owners who spent 107.1 min/week. Less time per week was spent in NTW than in NRW among dog-owners and non-owners (Table 2). Weekly minutes of NTW were not significantly different between dog-owners and non-owners, for either summer or winter. Adjusting for demographic characteristics, there was no statistically significant difference in the change in weekly NTW minutes from winter to summer between dog-owners and non-owners (Table 2). We did, however, find that dog-owners undertook on average 39.6 min/week **more **NWR in winter than in summer, while non-owners undertook 16.2 min/week **less **NWR in winter than in summer (Table. 2). This result suggests a difference between dog-owners and non-owners in weekly NWR minutes across seasons - even after adjusting for demographic characteristics.

**Table 2:** Comparison of usual weekly minutes walking for recreation and transportation inside the neighbourhood in summer and winter among dog-owners and non-owners

		Summer		Winter		Mean difference in minutes between winter and summer within dog-ownership status	
	**Dog ownership status**	**Mean(SD)**	**95%CI**	**Mean(SD)**	**95%CI**	**Mean difference**	**95%CI**

**Neighbourhood walking for recreation**	Non-owners	123.3 (157.7)	105.8,140.9*	107.1 (135.9)	92.0, 122.2*	16.2	-2.3, 34.8
	Owners	213.6 (206.8)	175.4, 251.8*	253.2 (211.8)	214.0, 292.3*	-39.6^†^	-70.2, -8.9
**Neighbourhood walking for transportation**	Non-owners	74.9 (123.7)	61.2, 88.7	69.8 (119.3)	56.5, 83.1	5.2	-7.4, 17.8
	Owners	59.1 (128.2)	35.4, 82.8	59.9 (112.6)	39.1, 80.7	-0.83	-21.6, 19.9

*Significant difference between dog-owners and non-owners within season (p < .05).

^† ^Change in minutes from winter to summer significantly different between dog-owners and non-owners (p < .05).

Negative values: mean walking minutes in winter > mean minutes of walking in summer.

Positive values: mean minutes of walking in summer > mean minutes of walking in winter.

All results adjusted for age, sex, ethnicity, marital status, income, education and type of house.

Among all respondents, 65.1% participated in NRW in both winter and summer, 8.9% participated in NRW in the summer only, 10.5% participated in NRW in the winter only, and 15.4% did not report any participation in NRW in the summer and winter (Table 1). Respondents who participated in NRW in the summer only (i.e., no NWR reported in winter) were non-owners. Adjusting for demographic characteristics, we found that dog-owners were at least 3 times more likely than non-owners to be consistent participators (i.e., in winter and summer) in NRW (OR = 3.28; 95%CI = 1.86, 5.79) (Table 3). Dog-owners were, in addition, less likely than non-owners to be inconsistent participators in NWR (OR = 0.35; 95%CI = 0.16, 0.79) (Table 3). Among all respondents, 43.7% participated in NTW in both winter and summer, 11.9% participated in NTW in the summer only, 12.8% participated in NTW in the winter only, and 24.8% reported no participation in NTW in summer and winter. Moreover, dog-owners were no more or less likely than non-owners to participate in NTW, in summer, in winter, or in both seasons (Tables 1 and 3).

**Table 3:** Odds ratios (OR) and 95% confidence intervals (95%CI) showing the association between walking for recreation and transportation inside the neighbourhood during a usual week (i.e., none vs. any) from winter to summer and dog ownership status

	Neighbourhood walking for recreation				Neighbourhood walking for transportation			
**Dog ownership status**	**Consistent participator**^1^	**Summer participator**^2†^	**Winter participator**^3^	**Consistent non-participator**^4^	**Consistent participator**^1^	**Summer participator**^2^	**Winter participator**^3^	**Consistent non-participator**^4^
	**OR (95%CI)**	**OR (95%CI)**	**OR (95%CI)**	**OR (95%CI)**	**OR (95%CI)**	**OR (95% CI)**	**OR (95%CI)**	**OR (95%CI)**

Non-owners	1.00	-	1.00	1.00	1.00	1.00	1.00	1.00
Owners	3.28 (1.86, 5.79)*	-	1.05 (0.49, 2.23)	0.35 (0.16, 0.79)*	0.65 (0.40, 1.05)	0.91 (0.45, 1.83)	1.44 (0.75, 2.74)	1.41 (0.86, 2.30)

* Participation significantly different between dog-owners and non-owners (p < .05)

^†^All respondents who participated in NWR during summer but not winter were non-owners.

^1^Walked in both summer and winter.

^2^Walked in summer only.

^3^Walked in winter only.

^4^No walking in summer and winter.

All results adjusted for age, sex, ethnicity, marital status, income, education and type of house.

## Discussion

Of the dog-owners in our sample, 63% reported walking their dog in both summer and winter; similarly, Australian research found that the majority of dog owners regularly walked their dogs [28]. Overall, dog-owners reported greater participation in NRW than did non-owners, supporting previous studies showing dog-owners to be more physically active than non-owners in recreational walking [11,19,29,30]. Yet our finding that dog-owner's participation in physical activity remained relatively stable from winter to summer is novel, as is the finding that dog-owners' NRW increased in winter, while non-owners' NRW decreased.

Overall, our study's longitudinal design allowed us to pose questions and draw conclusions that are not possible with cross-sectional data. To the best of our knowledge, research investigating dog-ownership as a factor in longitudinal patterns in neighbourhood walking has not previously been undertaken. Compared to non-owners, dog-owners walked more for recreation in both the winter and summer, which corresponds with previous studies that found dog-owners spend more time in mild to moderate physical activity, including walking [11,19,31] and in recreational physical activity [30]. Moreover, dog-owners' NWR, on average, was equivalent of 30-minutes/day regardless of season - the recommended minimum amount of physical activity to accrue health benefits [32]. In contrast, non-owners, on average, were not achieving this level of NWR, and in fact reported slightly less NWR in winter than in summer.

A significant difference in NTW between dog-owners and non-owners was not found in our study, but previous work has found that dog-owners are less likely to walk for transportation compared to non-owners [30]. The limited difference in NTW among dog-owners compared with non-owners could be attributable to a lack of dog-friendly parks, shops, and other commercial destinations within walking distance of home [33]. Allowing people to bring their dogs to work could be beneficial to owners as well as to non-owners [34], but dogs are not generally allowed in workplaces unless they are designated as service animals, such as seeing-eye dogs. Consequently, adults who walk to work generally leave their dogs at home. In addition, even though dogs are allowed on public transportation in the City of Calgary, the dog-owners in our sample who commute by public transit would have few options for dog care at or near businesses, workplaces, or educational institutions, thus impeding them from walking with their dog to bus stops or commuter train stations located within their neighbourhood.

This study contributes to the literature on determinants of physical activity by showing that dog-ownership contributes to walking for recreation in residential neighbourhoods despite seasonal fluctuations, yet has several limitations. While we used previously-tested items for capturing walking [26,27], self-reported physical activity is nevertheless affected by recall and memory bias [35]. The use of objective measures of physical activity (i.e., pedometers and accelerometers) in future research could overcome this limitation. About 45% of our original sample was lost to follow-up or was missing data, which may have introduced a bias to our sample [7]. The reasons why we found higher levels of NRW among dog-owners in the winter compared with the summer, furthermore, are not evident. One possibility is that during summer months owners and their dogs may be more likely to undertake recreational activities at destinations or in open spaces located outside their immediate neighbourhood (i.e., mountains, provincial parks, lakes) rather than close to home. Another possibility, is that in the summer months, dog-owners living in detached or semi-detached housing may be inclined to unleash their dogs in the backyard, rather than taking them out for walks in the neighbourhood. Future research using qualitative and quantitative methods is needed to better understand the interrelationships between dog-ownership, seasonality and physical activity behaviour.

## Conclusions

Health professionals should discuss but not prescribe dog-ownership as a way to promote physical and mental health via regular physical activity. Dog-ownership may provide a social support that encourages walking, therefore, initiatives to help shape a similar type of social support, such as dog-sharing programs, may help increase physical activity [36,37]. Furthermore, as not all dog-owners regularly walk their dog [28], destinations such as cafes, shops, and parks with dog-friendly attributes could encourage more walking among this group [38]. Dog-ownership status should be considered when designing and assessing interventions to increase year-round physical activity participation.

## Abbreviations

NRW: Neighbourhood-based recreational walking; NTW: Neighbourhood-based transportation walking.

## Competing interests

The authors declare that they have no competing interests.

## Authors' contributions

GRM and MR conceived the current study. PL led the drafting of the manuscript with input from GRM and MR. Data analysis was undertaken by PL, and was supervised by GRM. All authors contributed to the interpretation of the results. All authors read and approved the final manuscript.

## Pre-publication history

The pre-publication history for this paper can be accessed here:

http://www.biomedcentral.com/1471-2458/11/148/prepub
